# Ameliorative effects of Compound K and ginsenoside Rh1 on non-alcoholic fatty liver disease in rats

**DOI:** 10.1038/srep41144

**Published:** 2017-01-20

**Authors:** Xu-Jia Chen, Wen-Jing Liu, Meng-Liang Wen, Hong Liang, Shao-Mei Wu, Yun-Zhen Zhu, Jiang-Yuan Zhao, Xiang-Qian Dong, Ming-Gang Li, Li Bian, Cheng-Gang Zou, Lan-Qing Ma

**Affiliations:** 1 Yunnan Institute of Digestive Disease, Department of Digestive Diseases, First Affiliated Hospital, Kunming Medical University, Kunming, Yunnan 650032, China; 2 School of Medicine, Yunnan University, Kunming, Yunnan 650091, China; 3 Key Laboratory for Conservation and Utilization of Bio-resource in Yunnan, and Key Laboratory for Microbial Resources of the Ministry of Education, Yunnan Institute of Microbiology, Yunnan University, Kunming, Yunnan 650091, PR China; 4 Department of Pathology, First Affiliated Hospital, Kunming Medical University, Kunming, Yunnan 650032, China

## Abstract

Non-alcoholic fatty liver disease (NAFLD) is a common liver disease, which has no standard treatment available. Panax notoginseng saponines (PNS) have recently been reported to protect liver against hepatocyte injury induced by ethanol or high fat diet (HFD) in rats. Compound K and ginsenoside Rh1 are the main metabolites of PNS. In this study, we evaluated the effects of CK and Rh1 on NAFLD. Rats fed HFD showed significant elevations in liver function markers, lipids, glucose tolerance, and insulin resistance. Treatment with CK or Rh1 either alone or in combination dramatically ameliorated the liver function impairment induced by HFD. Histologically, CK and Rh1 significantly reversed HFD-induced hepatocyte injury and liver fibrosis. *In vitro* experiments demonstrated that treatment with CK or Rh1 alone or in combination markedly induced cell apoptosis, and inhibited cell proliferation and activation in HSC-T6 cells. Additionally, CK and Rh1, either alone or in combination, also repressed the expression of fibrotic factors TIMP-1, PC-I, and PC-III. Taken together, our results demonstrate that CK and Rh1 have positive effects on NAFLD via the anti-fibrotic and hepatoprotective activity.

Non-alcoholic fatty liver disease (NAFLD) refers to hepatic steatosis, or the accumulation of triglyceride in the liver, which is not caused by alcohol consumption^1^. It is one of the most common causes of chronic liver disease worldwide that increases liver-related mortality. The prevalence of NAFLD has been reported to be as high as 30% in western countries and 15% to 18% in Asia^2^,^3^. A recent meta-analysis has shown that the prevalence of NAFLD in China is approximately 20%^4^. This fatty liver disease is strongly associated with obesity. Considering current obesity epidemic, the incidence of NAFLD is expected to rise worldwide.

NAFLD includes a broad spectrum of diseases from simple hepatic steatosis to inflammatory steatoheapatitis (NASH) with increasing levels of fibrosis and ultimate cirrhosis^5^. The pathogenesis of NAFLD and its progression to fibrosis and chronic liver disease are still unknown. In 1998, Day *et al*.^6^ first proposed the “two-hit” hypothesis. The first hit is an initial metabolic alteration, such as insulin resistance, hyperglycemia, and the accumulation of triglyceride in hepatocytes, leading to hepatic steatosis. The second hit induces the progression to more severe injury including steatohepatitis, inflammation, fibrosis, and cirrhosis. In 2010, Tilg *et al*.^7^ proposed the “multiple parallel hits” hypothesis for NAFLD. According to this hypothesis, many parallel hits derived from the gut and/or the adipose tissue may promote liver inflammation. Endoplasmic reticulum stress and related signaling networks, cytokines, and innate immunity are emerging as central pathways that regulate key features of NASH.

Currently, the principal therapeutic modalities for NAFLD are lifestyle interventions^8^. However, pharmacologic treatments are limited. Many pharmacological treatments, such as insulin sensitizers, antioxidants and anti-inflammatory agents, are used for improving hepatic inflammation, fibrosis, and clearing steatohepatitis^9^. Herbal medicines have been reported to show protective effects on liver injury^10^,^11^,^12^. For instance, Sho-saiko-to is a common drug for treating chronic liver diseases in Asia^13^. Sho-saiko-to has been proven to effectively suppress liver fibrosis in rat models of fibrosis induced by a choline-deficient and amino acid-defined diet^14^, carbon tetrachloride^15^, and thioacetamide^16^.

*Panax notoginseng*, also known as Sanchi, has been used as an herbal medicine in China to stop bleeding, eliminate blood stasis, reduce swelling, and alleviate pain. The main active ingredients of *P. notoginseng* are panax notoginseng saponins (PNS). Previous studies have shown that PNS attenuate liver injury induced by high fat diet (HFD)^11^, lipopolysaccharide^17^ as well as ethanol in rats^18^,^19^. PNS also provide strong protection of hepatocytes from ischemic reperfusion injury in the early stage of transplantation^20^. A recent study has revealed that PNS also exhibit anti-fibrotic activities during liver fibrogenesis^21^. Additionally, PNS has been found to have many other pharmacological actions, such as anti-tumorigenic, anti-hypertensive, and anti-inflammatory activities^22^,^23^,^24^.

PNS are not absorbed by the digestive tract until they are metabolized by intestinal microflora after being taken orally. Ginsenosides Compound K (CK) and Rh1 are the main metabolites of PNS, which are absorbed into the systemic circulation^25^. CK and Rh1 are classified as triterpene saponins (Fig. 1a and b). CK has various biological activities including anti-carcinogenic, anti-inflammatory, anti-allergic, anti-diabetic, anti-aging, neuroprotective and hepatoprotective effects^26^,^27^. However, the effects of CK and ginsenoside Rh1 on hepatic injury have not been well studied. In this study, we evaluated the effects of CK and ginsenoside Rh1 on hepatic injury induced by HFD. We found that CK and ginsenoside Rh1 had anti-fibrotic and hepatoprotective activities in HFD-induced NAFLD.

## Results

### Effects on liver function and lipid metabolism

NAFLD rat model was successfully established after 10 weeks of HFD feeding. The levels of γ-glutamyl transpeptidase (γ-GT), alanine aminotransferase (ALT), aspartate aminotransferase (AST), and alkaline phosphates (ALP) in HFD-fed rats were markedly increased compared to control rats. These parameters were much reduced after treatments of HFD-fed rats with CK, Rh1 or both for 1 week (*P* < 0.05, Table 1). In contrast, Rosiglitazone, a drug known to be effective for NAFLD^9^, did not have any effects. These results suggest that CK and Rh1 ameliorate HFD-induced liver dysfunction.

Significant elevations in serum levels of total cholesterol (CHOL), free cholesterol (FCHOL), low density lipoprotein (LDL) and triglyceride (TG) were also observed in HFD-fed rats as compared to control rats (Table 1). Administration of CK or Rh1 either alone or in combination significantly reduced these parameters. The levels of high density lipoprotein (HDL) were lower in HFD-fed rats than in control rats. Treatments with CK or Rh1 either alone or in combination increased HDL levels to normal. Rosiglitazone did not show any effects. These results suggest that CK and Rh1 treatments improve HFD-induced abnormal lipid metabolism.

### Effects on insulin resistance

**Figure 2:**
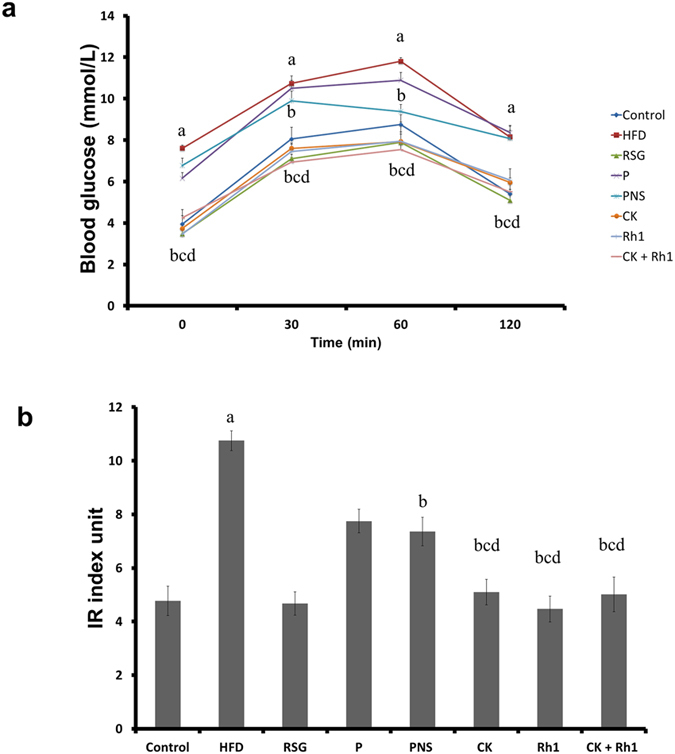
Effects of CK or Rh1 alone or in combination on insulin resistance in HFD-fed rats. (**a**) Blood glucose levels during GTT. (**b**) Insulin resistance index values between 0 and 120 min of GTT. Insulin resistance index = [fasting glucose (mmol/L) × fasting insulin (μU/ml)]/22.5. Control, normal diet alone; HFD, high fat diet alone; RSG, Rosiglitazone plus high fat diet; P, phospholipid plus high fat diet; PNS + P, the combination of panax notoginseng saponins and phospholipid plus high fat diet; CK + P, the combination of compand K and phospholipid plus high fat diet; Rh1 + P, the combination of Rh1 and phospholipid plus high fat diet; CK + Rh1 + P, the combination of CK, Rh1 and phospholipid plus high fat diet. n = 20. ^a^*P* < 0.05 versus control group, ^b^*P* < 0.05 versus HFD group, ^c^*P* < 0.05 versus phospholipid group, ^d^*P* < 0.05 versus PNS group.

Glucose tolerance tests (GTT) showed that serum glucose level reached a peak at 60 min and then declined in both control and HFD-fed rats, with glucose levels being always higher in HFD-fed rats than in the control rats. Treatments of HFD-fed rats CK, Rh1, or both brought the glucose levels to normal. As a positive control, Rosiglitazone also reduced glucose levels in HFD-fed rats (Fig. 2a). Insulin resistance index between 0 and 120 min was significantly higher in HFD rats than in control rats. However, when the HFD-fed rats were treated with Rosiglitazone, CK, Rh1 or the combination of CK and Rh1, the insulin resistance index was reduced to normal (Fig. 2b). These results suggest that Rosiglitazone, CK and Rh1 treatments alleviate HFD-induced insulin resistance.

### Effects on liver injury and fibrosis

**Figure 3:**
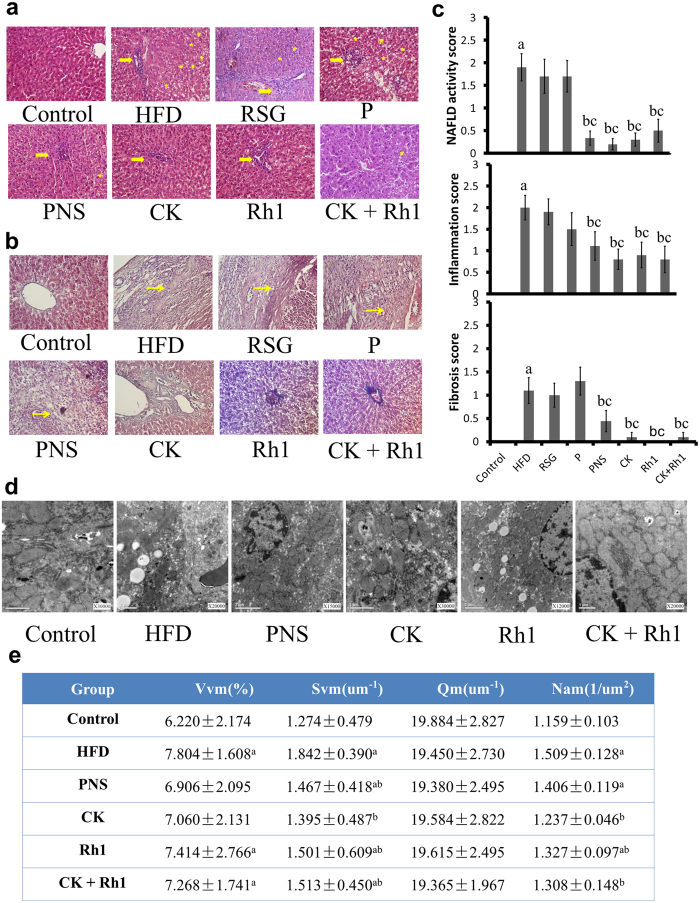
Effects of CK or Rh1 alone or in combination on liver structures in HFD-fed rats. (**a–c**) HE staining (**a**) and Masson’s trichrome staining (**b**) of liver sections are shown. Quantitative analysis of NAFLD activity, inflammation and fibrosis are shown (**c**). The thick yellow arrows represent inflammation. The asterisks represent steatosis, and the fine yellow arrows represent fibrosis. NAFLD activity score, inflammation score and the extent of liver fibrosis were graded by 4-grade scores. Three sections per liver were used for each animal and twenty mice were used for each group for staining. (**d** and **e**) Electron micrographs of liver sections (**d**) are shown. Quantitative analysis of mitochondia is presented (**e**). Data are the mean ± SD of thirty-eight independent graphs. Vvm: volume density of the mitochondrial; Svm: surface density of the mitochondrial; Num: Numerical density of the mitochondrial; Qm: Specific surface area of the mitochondrial. Control, normal diet alone; HFD, high fat diet alone; RSG, Rosiglitazone plus high fat diet; P, phospholipid plus high fat diet; PNS + P, the combination of panax notoginseng saponins and phospholipid plus high fat diet; CK + P, the combination of Compound K and phospholipid plus high fat diet; Rh1 + P, the combination of Rh1 and phospholipid plus high fat diet; CK + Rh1 + P, the combination of CK, Rh1 and phospholipid plus high fat diet. n = 20. ^a^*P* < 0.05 versus control group, ^b^*P* < 0.05 versus HFD group, ^c^*P* < 0.05 versus phospholipid group.

As determined by Hematoxylin and Eosin (HE) staining and Masson’s Trichrome staining, hepatic lobules were damaged; hepatic cords were arranged in disorder; and hepatocytes contained many vacuoles filled with lipid-droplets in the livers of HFD-fed rats (Fig. 3a and b). Meanwhile, fibrosis was found in some regions encircling hepatic lobules (Fig. 3b). In HFD-fed rats treated with Rosiglitazone, the majority of hepatic cells showed steatosis with minimal fibrosis. In HFD-fed rats treated with CK or Rh1 either alone or in combination, most of the hepatic cells were apparently normal without fibrotic lesions, and only a few scattered hepatic cells contained fewer and smaller vacuoles (Fig. 3a and 3b). Quantitative assessment of liver injury, inflammation and fibrosis in all the groups is presented in Fig. 3c.

Transmission EM demonstrated that ER was arranged in parallel, and were in close association with regularly distributed mitochondria containing well-organized cristae in control rats (Fig. 3d). In contrast, the livers of HFD-fed rats showed that an increase in lipid droplets, variations in shape and size of mitochondria, enlargement of mitochondria, and irregularity of mitochondrial cristae. In addition, swelling and fractures were often observed in the smooth ER. In HFD-fed rats treated with PNS, hepatocytes contained large fat droplets in perinuclear region, large-sized mitochondria, irregularity of mitochondrial cristae, and fractures in the ER. Administration of CK or Rh1 either alone or in combination reduced size and abundance of lipid droplets, increased the number of normal mitochondria, and improved the ER structure. Quantitative assessment of mitochondria is presented in Fig. 3e. These results suggest that CK and Rh1 treatments attenuate HFD-induced fatty liver and liver fibrosis.

### CK and Rh1 inhibited proliferation and induced apoptosis in HSCs

**Figure 4:**
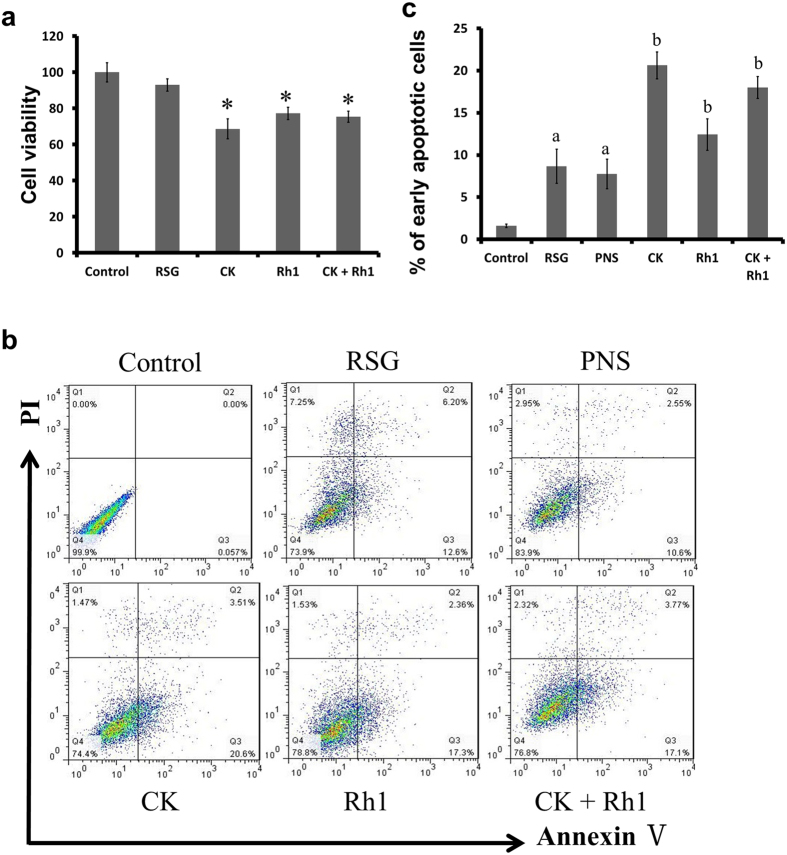
Effects of CK or Rh1 alone or in combination on cell proliferation and apoptosis in HSCs. (**a**) Cells were cultured in the presence of Rosiglitazone (RSG), CK, Rh1 or the combination of CK and Rh1 for 6 hours, before they were subjected to MTT assay. All results are the means ± SD of three independent experiments. *P < 0.05 versus control group. (**b**) Flow cytometric plots show cells in live, early apoptosis and late apoptosis when cells were treated with RSG, PNS, CK, Rh1 or the combination of CK and Rh1 for 6 hours. (**c**) Bar graphs show the percentages of early apoptotic cells. Data are the mean ± SD of three independent experiments. ^a^*P* < 0.05 versus control group, ^b^*P* < 0.05 versus PNS group.

HSC proliferation is a crucial step for hepatic fibrosis^28^. We thus tested the effects of CK and Rh1 on cell proliferation using MTT assay. Cells were incubated with Rosiglitazone, CK, Rh1 or a combination of CK and Rh1 for 6 hours. Compared with control cells, cell viability of HSC-T6 cells was reduced to 68.7%, 77.3% or 75.4%, by CK, Rh1 or both, respectively (*P* < 0.05) (Fig. 4a). Notably, Rosiglitazone did not alter the proliferation of HSC-T6 cells. However, CK and Rh1 treatments had a potent antiproliferative effect on HSC-T6 cells (*P* < 0.05).

In order to explore the pro-apoptotic effect of CK and Rh1 on HSC-T6 cells, flow cytometric analysis was performed to examine apoptosis. The proportion of apoptotic cells was 5% in control cells. When cells were treated with Rosiglitazone, PNS, CK, Rh1 or a combination of CK and Rh1, the percentages of early apoptotic cells were 8.65%, 7.73%, 20.63%, 12.43%, and 18%, respectively (Fig. 4b and c). These results indicate that CK and Rh1 induce apoptotic cell death in HSC-T6 cells.

### CK and Rh1 inhibited HSC activation

**Figure 5:**
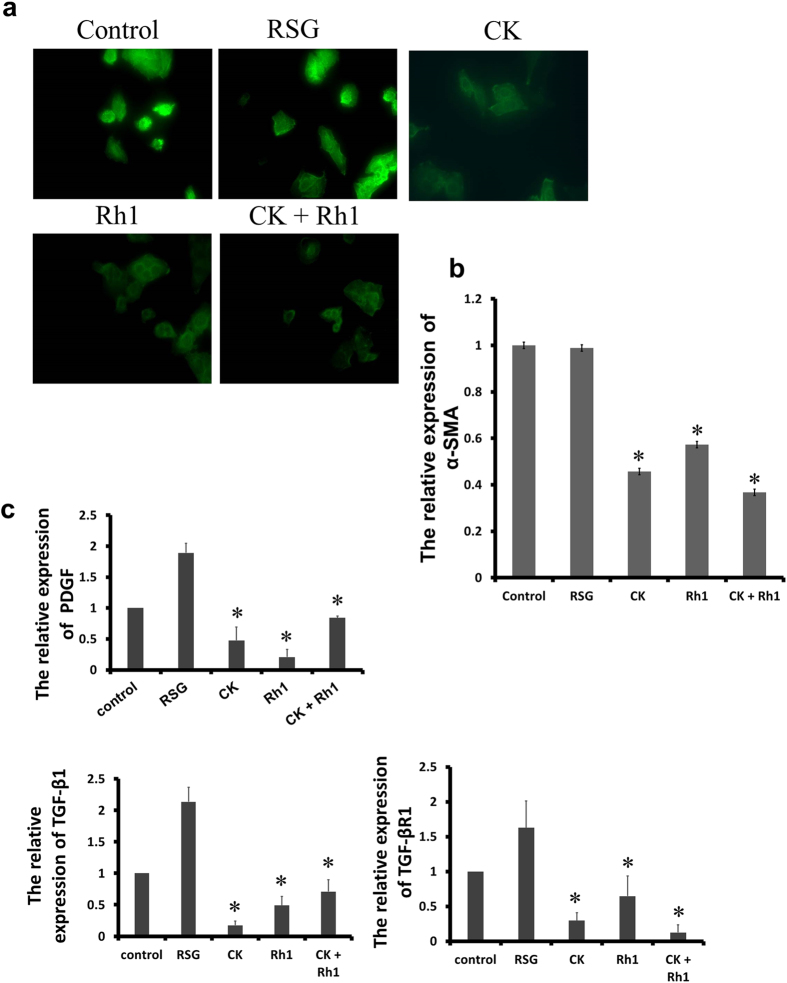
Effects of CK or Rh1 alone or in combination on cell activation in HSCs. (**a**) HSC-T6 cells were cultured in the presence of Rosiglitazone (RSG), CK, Rh1 or the combination of CK and Rh1 for 6 hours. Cells were then subjected to immunostaining for α-SMA. Original magnification ×400. (**b**) Quantitative analysis of the relative expression is shown. Data are the mean ± SD of three independent experiments. ^*^P < 0.05 versus control group. (**c**) Total RNA was extracted and subjected to real-time PCR. All results are normalized to the levels of β-actin and are the means ± SD of three experiments. ^*^P < 0.05 versus control group.

In the normal liver, HSCs are essentially quiescent, but have the ability to trans-differentiate into myofibroblast-like cells in response to liver injury during a process termed “activation”. Activation of HSCs plays a critical role in liver fibrogenesis. α-SMA is a marker for HSC activation. Using immunofluorescent staining with anti-α-SMA antibodies, we found that HSC-T6 cells showed high levels of α-SMA expression, indicating the cells were activated (Fig. 5a). No significant change in the expression of α-SMA was observed when treated with Rosiglitazone for 6 h. In contrast, there was a significant decrease in the expression of α-SMA when treated with CK or Rh1 either alone or in combination. Quantitative assessment of the relative expression of α-SMA is presented in Fig. 5b. These results suggest that CK and Rh1 suppress the activation of HSC-T6 cells.

HSC activation is coupled with increased expression of the profibrogenic proteins PDGF and TGF-β. As determined by real-time PCR, expression levels of PDGF, TGF-β1 and TGF-βR1 were increased by Rosiglitazone, but decreased by CK or Rh1 either alone or in combination (Fig. 5c). These results confirm that CK and Rh1 inhibit HSC-T6 cell activation.

### CK and Rh1 positively regulated the synthesis of extracellular matrix in HSCs

**Figure 6:**
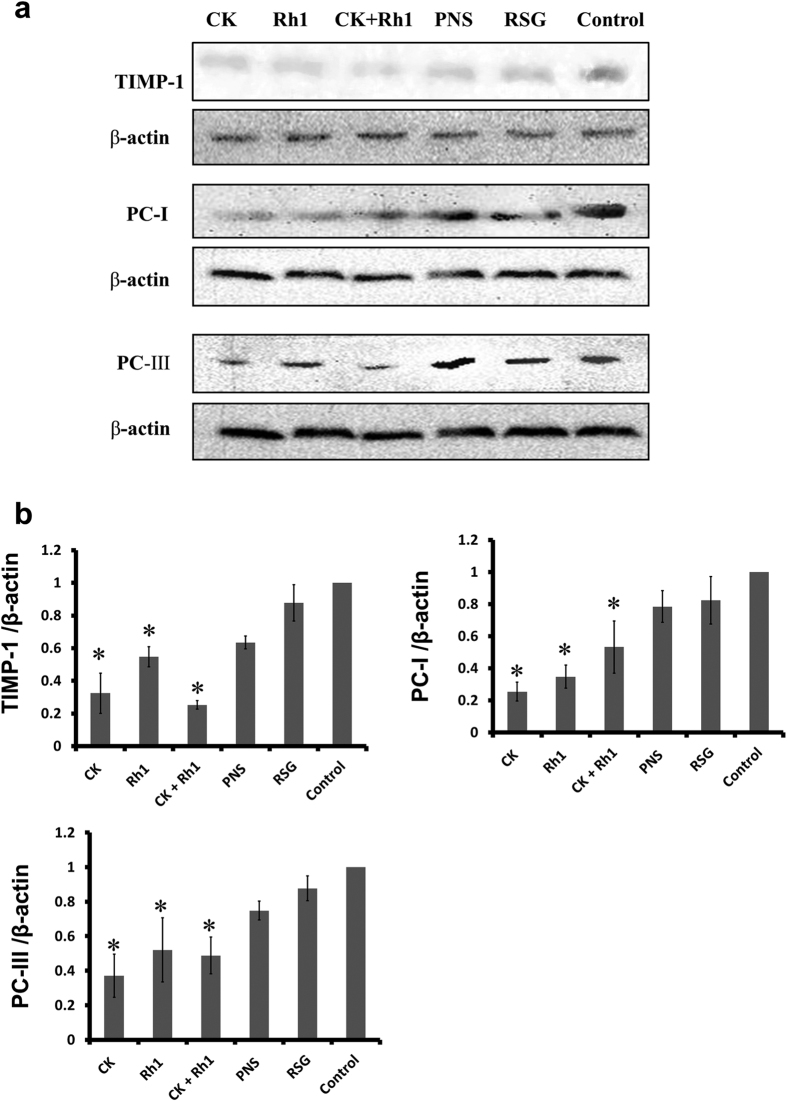
Effects of CK or Rh1 alone or in combination on the expression levels of fibrosis-associated proteins in HSCs. (**a**) Expression levels of TIMP-1, PC-I and PC-III in HSC-T6 cells were determined by Western blotting. β-actin was used as the loading control. Cells were cultured in the presence of Rosiglitazone (RSG), CK, Rh1 or the combination of CK and Rh1 for 6 hours. Representative blots show the expression of TIMP-1, PC-I and PC-III in different groups. (**b**) Bar graphs show the relative expression of TIMP-1, PC-I and PC-III in different groups. Data are the mean ± SD of three independent experiments. ^*^P < 0.05 versus control group.

Liver fibrosis is associated with the accumulation of ECM proteins. Procollagen (PC)-I, PC-III, and tissue inhibitor of metalloproteinases 1 (TIMP-1) are thought to play an essential role in the hepatic fibrosis^29^,^30^,^31^. To explore the mechanisms underlying the effects of CK and Rh1 on liver fibrosis, expression of ECM proteins was measured by Western blotting. Expression of PC-I, and PC-III and TIMP-1 was decreased when cells were incubated with CK or Rh1 either alone or in combination (Fig. 6a and b). These results further support an anti-fibrotic role of CK and Rh1 in the liver.

## Discussion

Previous studies have shown that PNS protect rats against hepatic injury induced by high fat diet^11^, lipopolysaccharide^17^ or ethanol^18^,^19^. PNS also suppressed hepatic fibrogenesis in Long-Evans rats with cinnamon coat color^21^. Although PNS are known to have hepatoprotective activity, the active ingredients are not yet fully identified. Several studies have shown that CK and ginsenoside Rh1 are the main metabolites of PNS^25^,^32^,^33^. In the present study, we have evaluated the effects of CK and Rh1 on liver injury and fibrosis using the widely used HFD-induced NAFLD rat model. Our data demonstrate that CK or Rh1 either alone or in combination, alleviates hepatic injury and insulin resistance, and improves liver fibrosis.

The HFD model is the most commonly used to explore the effects of herbal medicines on NAFLD. After eight weeks of HFD, mice had higher total serum cholesterol, triglyceride and transaminases levels as well as impaired glucose tolerance as compared to control mice^31^,^34^. As there are no approved therapeutic regimes for treatment of NAFLD, there is a pressing need to search for agents that ameliorate the phenotypes seen in NAFLD. Rosiglitazone is a recommended agent for short-term therapy for NAFLD, because it has anti-fibrotic effects by promoting cell apoptosis and reducing hepatic proliferation^9^. However, the use of Rosiglitazone has been severely restricted due to the increased occurrence of cardiovascular events and congestive heart failure. CK and Rh1 are generally considered to be safe and have minimal adverse effects. In our study, undesirable effects were not observed in rats treated with CK and Rh1 (data not shown). Thus, CK and Rh1 seem to be safe drugs for clinic use.

A large body of evidence supports a complex interaction between NAFLD and insulin resistance^35^,^36^. In our study, the levels of fasting glucose and insulin resistance index were higher in HFD rats than in control rats. Administration of CK or Rh1 alone or in combination reduced insulin resistance index and improved glucose tolerance.

Serum levels of the transaminases are reliable markers of hepatocellular damage. We found that administration of CK or Rh1 either alone or in combination lowered the concentrations of transaminases in HFD-fed rats. Moreover, CK and Rh1 treatments improved lipid accumulation by decreasing serum TG and TC. These results suggest that CK and Rh1 reduce liver injury and inhibit the progression of NAFLD in HFD-fed rats. The hepatoprotective effects are also evidenced by the histological improvement in liver steatosis, with decreases in the size and number of hepatic lipid-droplet vacuoles following CK and Rh1 treatments. Moreover, liver fibrogenesis in HFD-fed rats was ameliorated by administration of CK or Rh1 alone or in combination. These results suggest that CK and Rh1 may play a role in preventing hepatic fibrosis.

Liver fibrogenesis results from excessive deposition of extracellular matrix and is a part of the wound healing process triggered by activation of hepatic stellate cells^37^,^38^. The process is accompanied by cell necrosis, apoptosis and proliferation^39^. In our study, treatment of CK or Rh1 alone or in combination induced apoptosis and inhibited cell proliferation in HSCs. The activation of HSCs plays a critical role in the fibrogenesis. Our results demonstrate that CK and Rh1 suppressed the activation of HSCs. HSC activation is coupled with sequential overexpression of PDGF and TGF-β1^29^. PDGF is a potent mitogen for myofibroblasts, while TGF-β1 is a master regulator in the transformation of hepatic stellate cells to myofibroblasts. Due to the large number of myofibroblasts accumulated in fibrotic regions, the hepatic expression of α-SMA and type I/III collagen also increase significantly^31^. Previous studies have shown that PNS inhibit hepatic stellate cell activation and liver fibrosis via downregulating TIMP-1, PC-I, PC-III and TGF-β1 expressions^40^. Consistent with these previous findings, the present results showed that CK and Rh1 inhibited HSC activation and decreased PDGF and TGFβ1 expression. We also observed down-regulation of TIMP-1, PC-I, and PC-III by CK and Rh1, which may have resulted from the inhibition of TGF-β1 and PDGF expression by CK and Rh1. Therefore, our results indicate that CK and Rh1 suppress the activation and proliferation of HSCs, at least in part, by down-regulating the expression of TGF-β1 and PDGF.

In conclusion, CK and Rh1 have hepatoprotective and anti-fibrotic activities in NAFLD. Thus, CK and Rh1 may represent promising agents to reduce hepatic injury or liver fibrosis as a monotherapy or in combination. Additional studies are necessary to establish the efficacy and safety of CK and Rh1 regimens in clinical practice for patients with NAFLD.

## Materials and Methods

The study was approved by the Animal Care and Use Committee of Kunming Medical University. All experimental protocol including any relevant details were approved by the Animal Care and Use Committee of Kunming Medical University.

The methods Experiments on rats were carried out in accordance with the approved guidelines.

### Animals and experimental design

SD rats were obtained from the Animal Center, Kunming Medical University (Kunming, China). The animals were housed at a constant temperature of 22 °C, with a 12 hr light/dark cycle of 12:12 hours. Male rats (age, 8 weeks; body mass, 160 ± 10 g) were randomly divided into eight groups, with 20 rats in each group. Group 1 served as a control group and were fed *ad libitum* on normal rat chow diet throughout the experiment (11 weeks). The remaining seven groups were fed *ad libitum* on a HFD containing 87.7% standard diet (w/w), 10% pork fat (w/w), 2% cholesterol (w/w) and 0.3% bile salts (w/w)^41^ for 10 weeks. After one week, the rats on HFD were treated with the following regimens: group 2 (HFD group) received saline; group 3 (Rosiglitazone) received Rosiglitazone (4 mg/kg/day, i.p.); group 4 (Phospholipid) received phospholipid (30 mg/kg/day, i.p.); group 5 (PNS) received a combination of phospholipid and PNS (50 mg/kg/day, i.p.); group 6 (CK) received a combination of phospholipid and CK (3 mg/kg/day, i.p.); group 7 (Rh1) received a combination of phospholipid and Rh1 (3 mg/kg/day, i.p.); group 8 (CK + Rh1) received a combination of phospholipid, CK and Rh1 (3 mg/kg/day, i.p.). Ginsenoside CK and ginsenoside Rh1 were purified from *Panax notoginseng* or Ginseng by Yunnan Yunuo Biological Engineering Company limited. At the end of the experiments, rats were sacrificed. Serum and liver samples were collected and stored at −80 °C for further use.

### Histopathological examination

Tissues were collected from the same location of the livers. All samples were fixed in 4% paraformaldehyde, embedded in paraffin, stained with HE or Masson’s trichrome. The sections were examined under a light microscope and photographed with digital camera (Olympus). NAFLD activity score, inflammation score and the extent of liver fibrosis were graded by 4-grade scores^42^,^43^.

### Measurement of serum biochemical parameters

Serum γ-GT, ALT, AST, ALP, TG, CHOL, FCHOL, LDL and HDL were measured by an automated biochemistry analyzer (Hitachi 7060, Japan). For glucose tolerance tests (GTT), rats were fasted overnight. After baseline blood collection, rats were injected intraperitoneally with glucose (2 mg glucose/g body weight). Blood samples were taken from the tail of animals at 30, 60 and 120 minutes after glucose load. The blood glucose concentration was measured (OneTouch Ultra Glucometer; Lifescan, Milpitas, CA). Plasma insulin levels were determined by a radioimmunoassay kit (Linco Research, St Charles, MO). Insulin resistance was determined using the following formula: insulin resistance index = [fasting glucose (mmol/L) × fasting insulin (μU/ml)]/22.5.

### Cell culture and cytotoxicity test

The immortalized rat HSC line HSC-T6 was purchased from the Cell Bank, Kunming Institute of Zoology, CAS. HSC-T6 cells were grown in Dulbecco’s Modified Eagle Medium (DMEM, Gibco) supplemented with 10% Fetal Bovine Serum (FBS, Gibco). When cells were 60–80% confluent, the culture medium was changed to phenol-red free DMEM (Gibco) lacking FBS. Cells then were treated with CK and/or Rh1 as indicated.

For the MTT assay, cells were seeded in 96-well plates overnight, and treated with CK and/or Rh1 for the indicated durations. 20 μl MTT solution (Sigma) were then added into the medium at the concentration of 5 mg/ml for 4 hours. The formazan crystals were dissolved in 100 μl DMSO. Absorbance was measured at 570 nm in a microplate reader (Molecular Devices).

### Flow cytometry assay of apoptotic changes

Apoptosis was quantitated by flow cytometry after staining cells with annexin V-FITC/propidium iodide (PI) staining (BD). Briefly, after indicated treatments, cells were trypsinized and washed twice with cold PBS, and then resuspended in 1 × binding buffer with 5 μl annexin V and 5 μl of PI at a concentration of 5 × 10^5^/mL cells in a total volume of 100 μl. The cells were gently mixed and incubated in the dark for 15 minutes at room temperature before the number of apoptotic cells was quantified by flow cytometry (LSRFortessa, BD) within 1 hour.

### Quantitative real-time RT-PCR analysis

Total RNA from cells was isolated using TRIzol reagent (Invitrogen). Random-primed cDNAs were generated by reverse transcription of total RNA samples with SuperScript II (Invitrogen). Real-time PCR analysis was performed with the ABI Prism 7000 Sequence Detection system (Applied Biosystems) using SYBR^®^ Premix-Ex TagTM (Takara). All results were standardized to the levels of β-actin. The primers used for PCR were as follows:

PDGF: 5′-TTGTAACACCAGCAGCGTC-3′ (F),

5′-CCTCACATCTGTCTCCTCCT-3′ (R);

TGF-β1: 5′-GAAGGACCTGGGTTGGAAGT-3′ (F),

5′-GGTTGTGTTGGTTGTAGAGGG-3′ (R);

TGF-βR1: 5′-CAAACCACAGAGTAGGCACT-3′ (F),

5′-ATTTCCCAGAACACTAAGCCC-3′ (R);

β-actin: 5′-CAACTCCATCATGAAGTGTAAC-3′ (F),

5′-CCACACGGAGTACTTGCGCTC-3′ (R).

### Western blotting

After treatments, cells were lysed on ice for 30 min in lysis buffer (containing 0.15 M NaCl, 30 mM Tris, 1 mM phenylmethanesulfonyl fluoride, 1% Triton X-100, 1 mM EDTA, 10 μg/ml leupetin, 2 μg/ml pepstatin, 2 μg/ml aprotinin and 2 mM Na_3_VO_4_). A total of 20–40 μg of protein was separated by SDS-PAGE and transferred to polyvinylidene difluoride membranes. Primary antibodies were: anti-TIMP-1, anti-procollagen-I, anti-procollagen-III and anti-β-actin (Sigma). The secondary antibody was a peroxidase-coupled anti-goat IgG (GE Healthcare). The membrane was exposed to ECL Hyperfilm (GE Healthcare), and the film was developed. The bands were quantified by densitometry using Image J. Results were obtained from triplicate experiments.

### Immunofluorescent staining for α-SMA

HSC-T6 cells were seeded in 6-well plates and cultured in DMEM medium, then treated with Rosiglitazone, CK, Rh1 or the combination of CK and Rh1 for 6 hours. Cells were incubated with the primary antibody anti-α-SMA (Sigma) overnight at 4 °C. After three washes with PBS, the cells were incubated with fluorescence-conjugated secondary antibody (Sigma) at room temperature for 1 h. The cells were examined under a fluorescence microscope and photographed with digital camera (Leica). The relative expression of α-SMA was quantified by densitometry using Image J. Results were from triplicate experiments.

### Statistical analysis

Data were expressed as mean ± SD. Statistical difference between the groups was analyzed using one-way ANOVA, followed by Tukey’s multiple comparison. For nonparametric data, Mann-Whitney U test was used. A level of *P* < 0.05 was considered statistically significant.

## Additional Information

**How to cite this article**: Chen, X.-J. *et al*. Ameliorative effects of Compound K and ginsenoside Rh1 on non-alcoholic fatty liver disease in rats. *Sci. Rep.* **7**, 41144; doi: 10.1038/srep41144 (2017).

**Publisher's note:** Springer Nature remains neutral with regard to jurisdictional claims in published maps and institutional affiliations.

## Figures and Tables

**Figure 1:**
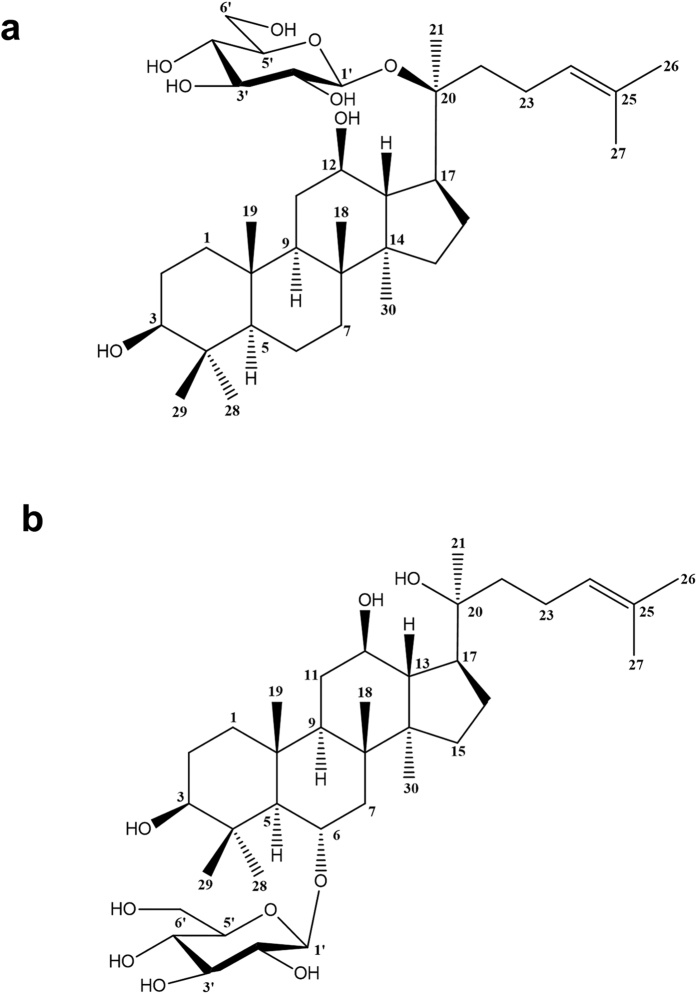
Chemical structures of CK and Rh1. (**a**) CK; (**b**) Rh1.

**Table 1:** Effects of CK or Rh1 alone or in combination on liver function and lipid metabolism in HFD-fed rats.

Group	Control	HFD	RSG	P	PNS	CK	Rh1	CK + Rh1
Weight after treatment (g)	284.88 ± 48.61	357.23 ± 20.56^a^	370.33 ± 24.92	370.90 ± 20.76	308.13 ± 32.33	296.52 ± 23.15	312.26 ± 19.81	331.27 ± 27.60
γ-GT (U/L)	0.22 ± 0.06	0.59 ± 0.06^a^	0.44 ± 0.06	0.62 ± 0.09	0.48 ± 0.10	0.23 ± 0.03^b,c,d^	0.29 ± 0.04^b,c,d^	0.32 ± 0.04^b,c,d^
ALT (U/L)	46.24 ± 15.53	92.52 ± 19.29^a^	69.22 ± 25.76	78.63 ± 43.67	72.92 ± 35.44	52.81 ± 8.70^b,c,d^	61.87 ± 18.87^b,c,d^	49.04 ± 9.12^b,c,d^
AST (U/L)	100.04 ± 28.48	318.92 ± 36.89^a^	211.37 ± 30.89	201.43 ± 38.58	182.34 ± 62.67	86.07 ± 16.9^b,c,d^	93.36 ± 9.10^b,c,d^	101.07 ± 17.04^b,c,d^
ALP (U/L)	108.46 ± 29.70	318.34 ± 47.95^a^	328.45 ± 43.26	443.63 ± 86.97	376.39 ± 154.19	155.69 ± 27.32^b,c,d^	131.56 ± 20.25^b,c,d^	129.19 ± 17.76^b,c,d^
CHOL (mmol/L)	1.52 ± 0.18	5.25 ± 2.30^a^	6.55 ± 2.04	8.23 ± 2.95	5.44 ± 2.11	2.50 ± 1.68^b,c,d^	3.87 ± 2.71^b,c,d^	3.15 ± 1.50^b,c,d^
FCHOL (mmol/L)	0.35 ± 0.07	1.33 ± 0.59^a^	1.89 ± 0.56	2.06 ± 0.72	1.58 ± 0.58	0.35 ± 0.40^b,c,d^	0.94 ± 0.64^b,c,d^	0.25 ± 0.37^b,c,d^
LDL (mmol/L)	0.51 ± 0.04	1.73 ± 0.78^a^	1.69 ± 0.86	2.81 ± 0.90	2.09 ± 0.74	0.52 ± 0.82^b,c,d^	1.00 ± 1.21^b,c,d^	0.73 ± 0.54^b,c,d^
HDL (mmol/L)	0.74 ± 0.08	0.37 ± 0.13^a^	0.35 ± 0.20	0.34 ± 0.24	0.40 ± 0.39	0.89 ± 0.19^b,c,d^	0.98 ± 0.25^b,c,d^	0.75 ± 0.15^b,c,d^
TG (mmol/L)	0.39 ± 0.06	0.72 ± 0.10^a^	0.68 ± 0.11	0.71 ± 0.13	0.74 ± 0.48	0.37 ± 0.07^b,c,d^	0.44 ± 0.13^b,c,d^	0.41 ± 0.15^b,c,d^
Fast insulin levels (mIU/L)	27.12 ± 1.15	32.79 ± 2.21	29.63 ± 1.49	30.85 ± 1.28	30.19 ± 2.35	30.29 ± 1.92	28.59 ± 1.62	29.71 ± 1.89

Values represent the mean ± SD of n = 20 rats/group. Rats in the normal diet (control) group were supplied with a normal rat chow diet for 11 weeks. Rats in the high fat diet (HFD) group were fed a high fat diet for 10 weeks first, and then fed a high fat diet plus Rosiglitazone (RSG), phospholipid (P), PNS, CK or Rh1 for 1 week. ^a^*P* < 0.05 versus control group, ^b^*P* < 0.05 versus HFD group, ^c^*P* < 0.05 versus phospholipid group, ^d^*P* < 0.05 versus PNS group. γ-GT: γ-glutamyltranspeptidase; ALT: alanine aminotransferase; AST: aspartate aminotransferase; ALP: alkaline phosphates. CHOL: total cholesterol; FCHOL: free cholesterol; LDL: low densitylipoprotein; HDL: high density lipoprotein; TG: triglyceride.
